# Effectiveness of spinal manipulation and biopsychosocial self-management compared to medical care for low back pain: a randomized trial study protocol

**DOI:** 10.1186/s12891-023-06549-w

**Published:** 2023-05-25

**Authors:** Gert Bronfort, Anthony Delitto, Michael Schneider, Patrick J. Heagerty, Roger Chou, John Connett, Roni Evans, Steven George, Ronald M. Glick, Carol Greco, Linda Hanson, Francis Keefe, Brent Leininger, John Licciardone, Christine McFarland, Eric Meier, Craig Schulz, Dennis Turk

**Affiliations:** 1 Integrative Health and Wellbeing Research Program Earl E. Bakken Center for Spirituality & Healing, University of Minnesota, Mayo Memorial Building C504, 420 Delaware Street, Minneapolis, MN 55414 USA; 2 School of Health and Rehabilitation Sciences, Department of Physical Therapy, University of Pittsburgh, 4029 Forbes Tower, Pittsburgh, PA 15260 USA; 3 School of Health and Rehabilitation Sciences, Department of Physical Therapy, University of Pittsburgh, Bridgeside Point 1, 100 Technology Drive, Suite 500, Pittsburgh, PA 15219 USA; 4 School of Public Health, Department of Biostatistics, University of Washington, 1959 NE Pacific Street, Box 357232, Seattle, WA 98195 USA; 5 School of Medicine, Division of General Internal Medicine, Oregon Health & Science University, 3181 SW Sam Jackson Park Road L475, Portland, OR 97239-3098 USA; 6 School of Public Health, Division of Biostatistics, University of Minnesota, 717 Delaware Street SE, 2nd Floor, Minneapolis, MN 5455 USA; 7 School of Medicine, Department of Orthopaedic Surgery, Duke University, 8020 North Pavilion, Durham, NC 27705 USA; 8 School of Medicine, Departments of Psychiatry and Physical Medicine & Rehabilitation, University of Pittsburgh, 580 S. Aiken Avenue, Suite 310, Pittsburgh, PA 15232 USA; 9 School of Medicine, Department of Psychiatry, University of Pittsburgh, 580 S. Aiken Avenue, Suite 310, Pittsburgh, PA 15232 USA; 10 School of Medicine, Department of Medicine, Duke University, 2200 W Main St., Suite 340, Durham, NC 27705 USA; 11 Health Science Center, University of North Texas, 3500 Camp Bowie Blvd, Fort Worth, TX 76107 USA; 12 School of Public Health, Department of Biostatistics, University of Washington, 4333 Brooklyn Avenue NE, Box 359461, Seattle, WA 98195 USA; 13 Department of Anesthesiology & Pain Medicine, University of Washington, Box 358045, Seattle, WA 98195 USA

**Keywords:** Low back pain, Spinal manipulation, Medical care, Self-management

## Abstract

**Background:**

Chronic low back pain (cLBP) is widespread, costly, and burdensome to patients and health systems. Little is known about non-pharmacological treatments for the secondary prevention of cLBP. There is some evidence that treatments addressing psychosocial factors in higher risk patients are more effective than usual care. However, most clinical trials on acute and subacute LBP have evaluated interventions irrespective of prognosis.

**Methods:**

We have designed a phase 3 randomized trial with a 2 × 2 factorial design. The study is also a Hybrid type 1 trial with focus on intervention effectiveness while simultaneously considering plausible implementation strategies. Adults (n = 1000) with acute/subacute LBP at moderate to high risk of chronicity based on the STarT Back screening tool will be randomized in to 1 of 4 interventions lasting up to 8 weeks: supported self-management (SSM), spinal manipulation therapy (SMT), both SSM and SMT, or medical care. The primary objective is to assess intervention effectiveness; the secondary objective is to assess barriers and facilitators impacting future implementation. Primary effectiveness outcome measures are: (1) average pain intensity over 12 months post-randomization (pain, numerical rating scale); (2) average low back disability over 12 months post-randomization (Roland-Morris Disability Questionnaire); (3) prevention of cLBP that is impactful at 10–12 months follow-up (LBP impact from the PROMIS-29 Profile v2.0). Secondary outcomes include: recovery, PROMIS-29 Profile v2.0 measures to assess pain interference, physical function, anxiety, depression, fatigue, sleep disturbance, and ability to participate in social roles and activities. Other patient-reported measures include LBP frequency, medication use, healthcare utilization, productivity loss, STarT Back screening tool status, patient satisfaction, prevention of chronicity, adverse events, and dissemination measures. Objective measures include the Quebec Task Force Classification, Timed Up & Go Test, the Sit to Stand Test, and the Sock Test assessed by clinicians blinded to the patients’ intervention assignment.

**Discussion:**

By targeting those subjects at higher risk this trial aims to fill an important gap in the scientific literature regarding the effectiveness of promising non-pharmacological treatments compared to medical care for the management of patients with an acute episode of LBP and the prevention of progression to a severe chronic back problem.

**Trial registration:**

ClinicalTrials.gov Identifier: NCT03581123.

## Background

The United States is in the midst of an unprecedented pain management crisis [1] with annual costs estimated at $560 to $635 billion per year [2]. Low back pain (LBP) is the most common chronic pain condition in adults and one of the leading causes of disability worldwide [3, 4]. Approximately 20% of acute cases will become chronic, [5] with roughly 40% of those with chronic LBP (cLBP) experiencing high-impact pain that significantly interferes with work, social activities, and daily life [6–8]. Given the socio-economic consequences of high-impact cLBP, research focusing on its prevention has become a national imperative [7–11].

It is now widely recognized that LBP is a complex condition influenced by several interrelated physical, psychological, and social factors [12]. However, most treatments still focus entirely on symptom management using a ‘one size fits all’ approach that fails to address the biopsychosocial (BPS) needs of those affected [13–17]. Treatment is frequently characterized by the persistent use of marginally effective and potentially harmful therapies that largely ignore the psychosocial aspects of LBP. For example, the use of epidural injections, opioid prescriptions, and spinal surgeries for LBP has increased at accelerating rates over the past few decades with little positive impact on patient outcomes [18, 19]. Of particular concern is the overreliance on opioids, which are used by an estimated 30% of chronic LBP patients [20] despite LBP clinical guidelines suggesting other pharmacologic and nonpharmacologic treatment options [21] and mounting recognition of opioid misuse, addiction, and fatal overdose [1].

There is growing evidence that physical and psychosocial risk factors can predict whether or not acute LBP progresses to become chronic [5, 22]. Such evidence has led to recommendations for clinical trials to focus on participants at higher risk of chronicity, limiting the testing of interventions to those most in need [23]. There is also evidence that treatments addressing psychosocial risk factors in patients at risk for chronicity are more effective than treatment with usual care [24]. However, most clinical trials to date on acute and subacute LBP populations have tested interventions irrespective of prognosis, limiting the ability to make confident conclusions about their effectiveness in terms of secondary prevention among high-risk subjects [23]. Consequently, there is a need for research that can more rigorously assess the potential of promising interventions to prevent acute and sub-acute LBP from progressing to more persistent severe cLBP by appropriately targeting those at higher risk.

To reduce cLBP burden, patients should have greater access to front-line care addressing both their physical and psychosocial needs. To accomplish this, there has been increased interest in studies of multi-modal interventions that are better suited to meet patients’ whole person needs [25]. Such approaches are designed to integrate psychosocial interventions with traditional biologically based pain management approaches [26]. Physical therapists (PTs) and chiropractors (DCs) are the most common providers of non-pharmacologic treatment for LBP in the United States, with approximately 39% of LBP patients seeking treatment from DCs and 34% from PTs [27]. Both PTs and DCs help patients manage symptoms and aid in the restoration of movement and functional ability. Therefore, they are well suited to integrate psychosocial and biophysical strategies, [26, 28] and play an essential role in the frontline management of patients with LBP [29, 30].

Our long-term objective is to reduce overall LBP burden and prevent acute and sub-acute LBP from progressing to a severe chronic back problem, by targeting those at higher risk. We will assess the effectiveness of first-line non-pharmacologic strategies that address patients’ biopsychosocial needs compared to front-line medical care that consists of primarily pharmacological management.

## Methods/design

The purpose of this manuscript is to describe the design and methods of the PACBACK (**P**reventing **A**cute to **C**hronic **Back** Pain) trial (GRANT # UG3AT008769 and UH3AT008769) in accordance with the SPIRIT and CONSERVE guidance [31, 32]. The trial is a two-site, prospective, parallel group, phase 3 randomized type I hybrid effectiveness-implementation trial with a 2 × 2 factorial design. Adults with acute or subacute LBP and who are at moderate to high risk of chronicity, will be randomized to one of 4 interventions: supported self-management (SSM), spinal manipulation therapy (SMT), both SSM and SMT (SSM + SMT), or medical care (MC). Treatment duration will be up to 8 weeks. The trial is being conducted at University of Minnesota (UM) and University of Pittsburgh (UP) affiliated research clinics, with the UP serving as the central IRB (Institutional Review Board).

The first phase of PACBACK took place from September 2017 to November 2019 and included activities such as securing regulatory approvals, performing cross-site training of study staff and providers, developing study protocols and manuals of operations, and establishing data safety and monitoring and study accrual and retention plans. Additionally, the initial phase included an enrollment and randomization of 92 participants to assess performance milestones related to recruitment strategies, enrollment rates, intervention adherence and fidelity, and data collection. Upon satisfactory attainment of the performance milestones, a transition was made to the second phase, which started in November 2019 and is currently in active enrollment. The total sample size goal is 1,000 participants including the 92 participants from the initial phase.

### Trial objectives

The primary objective of the trial is to determine intervention effectiveness by assessing average low back pain and disability over 12 months post-randomization, and prevention of progression to severe cLBP at 10–12 months follow-up. Our hypotheses are informed by our prior research [33–50]. We hypothesize that SMT and SSM will both be effective relative to MC, and that SSM + SMT will have a partially additive effect and therefore be more effective than either SMT or SSM alone.

Secondary objectives are to explore implementation related factors by gathering data from participants, clinicians and other stakeholders to inform future implementation, including the novel SSM intervention, if warranted by the trial results [51].

### Roles and responsibilities

UM and UP serve as the Clinical Coordinating Centers (CCC) which oversee recruitment, screening and treatment in Minneapolis/St. Paul and Pittsburgh. The University of Washington (UW) serves as the Data Coordinating Center (DCC) and oversees auditing of trial conduct. Central IRB approval has been granted through UP (PRO18010414). An independent Data Safety and Monitoring Board (DSMB) and the funder, National Center for Complementary and Integrative Health (NCCIH), monitors project progress and reviews and approves all significant protocol amendments prior to implementation.

### Tools and framework

**Fig. 1:**
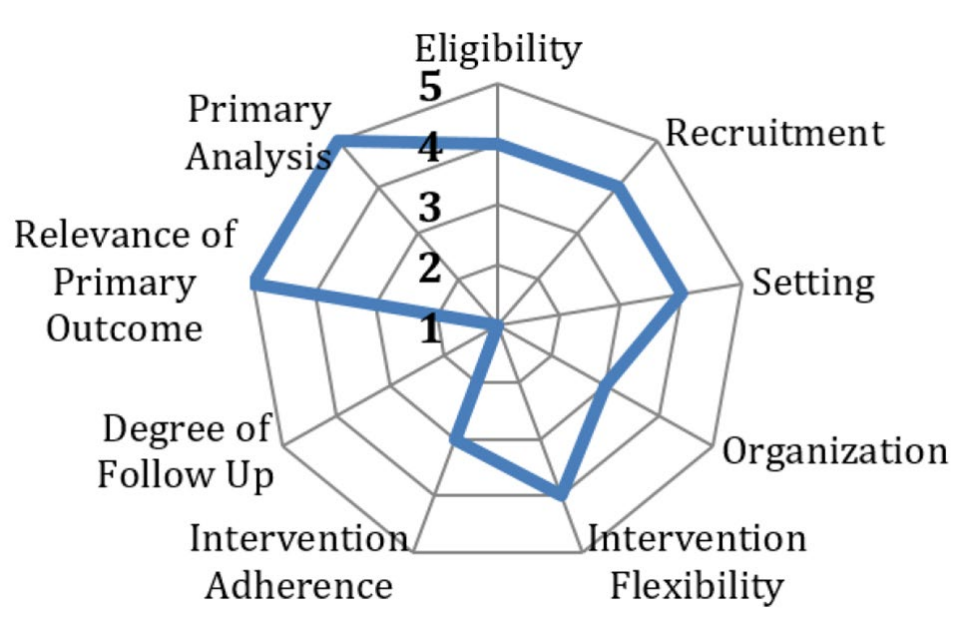
PACBACK trial mapped to the domains of the PRECIS-2 tool^26^ (Scores:5 = fully pragmatic,1 = fully explanatory)

The PRECIS-2 (Pragmatic Explanatory Continuum Indicator Summary) tool has been used to guide the study design and provide clarity regarding the pragmatic and explanatory features of the study (Fig. 1) [52]. The project is also informed by the RE-AIM framework which has provided guidance to addressing critical contextual factors related to Reach, Effectiveness, Adoption, Implementation and Maintenance, that can affect long-term implementation of the interventions [51].

### Recruitment

We are using a multi-faceted recruitment campaign to reach potential participants from the general public. Strategies include: social media (e.g., Facebook, Instagram, Twitter) and direct mail distribution to zip codes with racial and ethnically diverse populations; mass transit billboard advertising; digital advertising (e.g., Google and local media focused on reaching Persons of Color); community engagement activities (e.g., volunteering, providing health related presentations, tabling at local events, participating in local radio shows); distribution of study flyers; electronic advertisements in University affiliated health clinics; *ResearchMatch* and University research registries; and other routine university communication platforms (e.g., podcasts and newsletters).

### Screening and eligibility

Participants are initially screened by internet-based survey or phone, followed by a more in-depth phone screen with a clinician (PT, DC, nurse practitioner or physician assistant) trained in the study protocols. Willing and eligible individuals are then scheduled for a baseline screening appointment that includes informed consent and a health history and physical examination conducted by a clinician. Screening is followed immediately by a review of findings by the clinician with a study investigator (by phone) who together determine the eligibility of the participant based on the inclusion and exclusion criteria (see Table 1). The examining clinician follows a standardized algorithmic interview process in determining participant study eligibility. To qualify, participants must have experienced a new episode or aggravation of ongoing LBP in the past 12 weeks that lasted at least 2 weeks with a low back pain rating of three or more on average in the week before the baseline appointment. In addition, participants experiencing an aggravation of their LBP must rate their LBP in the month prior to the aggravation as mild or moderate, but not severe. Further, individuals with an aggravation of their LBP must agree that it is a worsening of their condition that is difficult to tolerate and generally impacts their usual activities and/or emotions. This operational definition is informed by an international consensus project that incorporated both patient and expert views for defining an aggravation or ‘flare’ of LBP [53].

**Table 1:** Inclusion and exclusion criteria for the study

Inclusion Criteria	Exclusion Criteria
18 years of age or older	Average LBP characterized as severe in the month preceding the current episode/aggravation
At the time of randomization, the participant’s current episode/aggravation of LBP must be between 2 and 12 weeks in duration.	Specific non-mechanical causes of LBP
Participants less than severe LBP on average in the month prior to the current episode/aggravation are eligible.	Contraindications to SMT or SSM (e.g., spinal fracture, progressive neurological deficits, inflammatory arthropathies of the lower back, surgical fusion of lumbar spine).
Average LBP severity ≥ 3 on the 0–10 numerical rating scale over past 7 days	Active management of current episode of LBP by another healthcare provider. Participants must agree to stop management with their current provider to enroll in the study (e.g., SMT, PT, prescription medication, psychological counseling/therapy, a structured program led by a healthcare provider that may include pain education, mind-body practices, coping strategies) to be included. Participants who had been prescribed opioid medication for LBP are required to obtain a note from a prescribing/medical provider to confirm they have safely discontinued their use of opioid medication to be included in the trial.
Medium or High Risk for persistent disabling back pain according to the STarT Back screening tool	Serious comorbid health condition that either requires medical attention (e.g., severe hypertension, inadequately managed serious mental health conditions, substance abuse), or has a risk for general health decline over the next year (e.g., Parkinson’s disease, Multiple Sclerosis, organ failure, Dementia, Alzheimer’s disease).
Ability to read and write fluently in English	Pregnancy, current or planned, and nursing mothers during the study period.
	Inability or unwillingness to give written informed consent.

LBP = Low Back Pain; PT = Physical Therapy; SMT = Spinal Manipulation Therapy; SSM = Supported Self-Management

### Randomization

The DCC administers and maintains the centralized randomization system. Randomization is stratified by site and baseline STarT Back screening tool risk status (medium risk, or high risk of cLBP) [22, 54]. Within strata block randomization is applied using variable block sizes of 8 or 12 participants. Allocation is concealed from all CCC investigators and staff by centralized randomization, and the number of participants previously randomized to each group is concealed from the study personnel involved in eligibility determination.

### Blinding

Blinding of clinicians and participants is not feasible due to the nature of the interventions. To minimize potential bias, all study personnel involved in screening and enrollment are masked to group assignments. Further, study personnel performing objective assessments are independent of intervention delivery and will remain blinded to study assignment until the end of data collection. One DCC staff member is unblinded and can access group assignment for closed DSMB reports. Additionally, all participants are queried in self-report questionnaires regarding any perceived attempts to influence their responses.

### Interventions

Eligible participants are randomly assigned to one of four interventions: Spinal Manipulation Therapy (SMT), Supported Self-Management (SSM), a combination of SSM and SMT (SSM + SMT), and Medical Care (MC). The intervention design has been informed by previous research, [33–50] qualitative work of patients’ perspectives, [55, 56] and discussions with clinical providers and researchers. The interventions are provided face to face at outpatient research clinics affiliated with UP and UM or via video-conferencing technology using HIPAA-compliant Zoom. Descriptions of the interventions following the Template for Intervention Description and Replication (TIDieR) are provided in Table 2 and summarized below. The following are common across the intervention groups:

- The intervention period can last up to 8 weeks.
- Interventions are provided by licensed clinicians with a minimum of three years of clinical practice experience; all clinicians receive study specific training (see Table 2) and are provided a manual of operations.
- Patients in all 4 groups receive standardized evidence-based information using a Back In Action booklet that describes the generally favorable prognosis of acute and sub-acute LBP, and simple strategies for remaining active and managing pain. All providers promote self-care practices consistent with the information in this booklet.
- Patients can continue with their routine self-administrated over-the-counter pain management medications and self-care activities (which is measured monthly along with other healthcare use as described under Outcome Measures).
- Only participants randomized to the medical care group received prescription medication. However, patients who experience a significant worsening of LBP symptoms during the 8-week intervention period in the SMT and SSM groups that requires additional management are referred to one of the trial medical providers for a short course of ‘rescue medications’, using a protocol from previous studies by the investigators [36, 37].
- During the 12-month follow-up period participants who experience a recurrence of an acute LBP episode (if still meeting the study inclusion/exclusion criteria) are given the option to receive a short course of additional care in the group they were originally assigned.

### Prohibited interventions

Participants are asked to limit treatment to their assigned intervention for the length of the initial 8-week intervention period; similarly, providers have been trained to refrain from delivering interventions that fall outside the scope of the study protocols. Participants retain the right to discontinue care at any time.

### Supported self-management (SSM)

SSM is provided by licensed PTs (n = 4) and DCs (n = 4). The design of the SSM intervention was guided by the Behavior Change Wheel model (BCW), coupled with the dynamic biopsychosocial model of pain that acknowledges the complex and reciprocal interactions between the evolving biopsychological or “whole” person and their external, social environment [57–59]. SSM aims to provide patients the opportunities and resources to develop their capacity and motivation to self-manage their LBP [59]. It entails 4–8, 60-minute sessions. The main intervention elements include a biopsychosocial oriented needs assessment; individualized treatment plan; education and training in physical and mind-body exercises and strategies; empowerment and support; and persuasion. Additional details related to the SSM intervention are provided in Table 2; the design and development of SSM will also be described in a separate manuscript.

**Spinal Manipulation Therapy (SMT)**. The SMT intervention is provided by licensed PTs and DCs. It is comprised of a minimum of two, 15–20 min visits and includes manipulative techniques with sufficient flexibility to be representative of the professions most commonly delivering SMT. The primary goal of the SMT intervention is to address the biophysical aspects of LBP with a focus on restoring spinal movement and functional ability. SMT includes a biophysical oriented needs assessment and development of an individualized treatment plan. The number of visits, spinal levels treated, and choice of SMT and supportive modalities are determined by provider according to patient needs and tolerance. SMT, includes grades 1–4, mobilization (low velocity, low-high amplitude passive movements) and/or manipulation (high velocity, low amplitude thrust) to the spine between the fifth thoracic and fifth lumbar vertebrae, and the sacroiliac joints. The chosen SMT techniques are based on those used in the UK BEAM Trial [60] and agreed upon by PT and DC professional groups. Supportive modalities including soft tissue techniques (cross-fiber stretch, longitudinal stretch, direct pressure, and deep friction massage), lumbar neural mobilization, and up to 10 min of heat.

**Supported Self-Management (SSM)** plus **Spinal Manipulation Therapy (SMT)**. The SSM + SMT intervention is provided by licensed PTs and DCs. It involves a minimum of four, 75–80 min visits and includes the modalities and strategies described for SSM and SMT above.

**Medical Care (MC)**. The MC intervention is provided by licensed physicians or advanced practice providers and entails a minimum of 2, 15–30 min visits during which patients receive guideline-based medication management, [21] which is a standard first-line approach for LBP in primary care. The primary goal of the MC intervention is to manage patient pain symptoms and restore daily function. MC includes review of symptoms, health history, and concomitant medications; medication choices are made based on clinician judgement and patient preferences. The first visit occurs in person or via videoconference; subsequent visits occur in person, via videoconference, or by phone as is standard in clinical practice. Decisions regarding visit frequency are made collaboratively by the provider and patient. Prescribed medications, include nonsteroidal anti-inflammatory drugs (NSAIDs, oral or topical) and skeletal muscle relaxants as a first-line approach. Patients who are unresponsive or unable to tolerate first-line medications may be prescribed acetaminophen, lidocaine patches, opioids, benzodiazepines, antiseizure medications, antidepressants, and selective serotonin reuptake Inhibitors and/or serotonin norepinephrine reuptake inhibitors. If deemed necessary providers have the option to recommend the use of heat, massage, or acupuncture; however, no formal referrals will be made.

**Table 2:** Description of Interventions based on the Template for Intervention Description and Replication (TIDieR [61])

	SSM	SSM + SMT	SMT	MC
**Rationale and Goal**	**Rationale**: LBP is a biopsychosocial (BPS) condition; front-line providers like PTs and DCs are well suited to provide support that will encourage adaptive self-management behaviors using multiple BPS-oriented evidence-based modalities **Goal**: to address the BPS aspects of LBP in an individualized manner so patients have what they need to effectively self-manage	**Rationale**: as described for SSM and SMT **Goals**: as described for SSM and SMT	**Rationale**: SMT is recommended by evidence-based guidelines for LBP; it is a modality used by common front-line providers like PTs and DCs **Goal**: to address the physical/biological aspects of LBP in an individualized manner, to restore spinal movement and functional ability	**Rationale**: Medications are recommended by evidence-based guidelines for LBP; they are used by common front-line providers like physicians and advanced practice providers; it is well suited to serve as a pragmatic comparison intervention **Goal**: to reduce pain symptoms as it would typically be delivered in primary care settings
**Participant materials**	-Back In Action booklet -Print workbook summarizing education and training procedures **-**Audio recordings of progressive muscle relaxation and guided imagery	-Includes patient materials as described for SSM and SMT	-Back In Action booklet	-Back In Action booklet
**Clinician materials**	-Manual of operations -Clinician guide with session checklists and other prompts and cues (e.g., suggested language, things to watch for) to facilitate delivery of essential procedures	-Includes clinician materials as described for SSM and SMT	-Manual of operations	-Manual of operations
**Procedures**	-Needs assessment, individualized treatment plan (also see Tailoring and Individualization below) -Education related to pain physiology, mind-body connection, etc. -Training in physical exercises (postural, strength, stabilization and mobility); psychological ‘mind-body’ strategies (relaxed breathing, progressive muscle relaxation, guided imagery, cognitive restructuring); and social strategies (pleasant activity planning and communication techniques for navigating social roles) -Empowerment and support to enhance facilitators and reduce barriers through goal setting and review; problem solving; action planning; general and emotional social support -Persuasion using patient-centered communication to foster therapeutic alliance	-Includes needs assessment, individualized treatment plan, and procedures as described for SSM and SMT	-Needs assessment, individualized treatment plan (also see Tailoring and Individualization below) -Spinal manipulation (Grades 1–4 mobilization, manipulation) -Supportive modalities (soft-tissue techniques, lumbar neural mobilization, heat)	-Needs assessment, individualized treatment plan -Medications (1st line: NSAIDs, skeletal muscle relaxants; 2nd line: acetaminophen, lidocaine patches, benzodiazepines, antiseizure medications, tricyclic antidepressants and selective serotonin reuptake inhibitors and/or serotonin norepinephrine reuptake inhibitors -Supportive modalities (heat, recommendations for massage, and acupuncture)
**Clinicians and Training**	-Physical therapists, chiropractors -minimum 3 years of experience -20 hours initial training; monthly 1 hour group clinician meetings to facilitate fidelity; additional refresher training as needed	-Physical therapists, chiropractors -minimum 3 years of experience -includes training as described for SSM and SMT	-Physical therapists, chiropractors -minimum 3 years of experience -4 hours initial training; monthly 1 hour group clinician meetings to facilitate fidelity; additional refresher training as needed.	-Medical physicians, nurse practitioners -minimum 3 years of experience -4 hours initial training; monthly 0.5-hour group clinician meetings to facilitate fidelity; additional refresher training as needed.
**Format**	-One-to-one -In person or via videoconference	-One-to-one -In person or via videoconference (SSM portion only)	-One-to-one -In person	-One-to-one -In person or via videoconference or telephone (after 1st visit)
**Tailoring and individualization**	-Number and frequency of visits depends on needs after minimum of 4 reached; determined by Self-Reliance check in assessing confidence in self-management, and Wellbeing Wheel -Education: information reiterated, and supplemental sleep, communication and physical activity information presented if indicated -Training: home exercise plan including practice of physical exercises and psychological ‘mind-body’ strategies tailored to needs, goals and abilities -Empowerment/support: customized to patients needs related to training goals and general and emotional support. -Persuasion: communication based on patient needs for to stimulate action -Additional emphasis on information from Back in Action booklet per individual needs	-Includes tailoring as described for SSM and SMT	-Frequency and number of visits after minimum of 2 reached. -Spinal levels treated, choice of grades 1–4 mobilization and/or manipulation based on clinical presentation, patient tolerance. -Additional emphasis on information from Back in Action booklet per individual needs	-Frequency and number of visits after minimum of 2 reached. -Medication(s) prescribed based upon participant’s prior history and preferences and clinician judgment. -Additional emphasis on information from Back in Action booklet per individual needs
**Frequency, Duration**	-4-8 visits over 8 weeks -up to 60 min	-4-8 SSM and ≥ 2 SMT visits over 8 weeks -up to 60 min	-≥ 2 visits over 8 weeks -15–20 min	-≥ 2 visits over 8 weeks -30 min
**Locations**	Physical Therapy Clinical and Translational Research Center (PT-CTRC) in Pittsburgh, PA, and the Epidemiology Clinical Research Center and Berman Center for Outcomes and Clinical Research in Minneapolis, MN. HIPAA compliant telemedicine SSM and MC sessions allowed prior to, during, and after COVID restrictions.			
**Modifications**	-Enrollment to two of the interventions (SSM + SMT, SMT) suspended temporarily due to COVID impacts on in-person clinic activity; other two interventions (SSM, MC) transitioned to videoconference delivery until in-person clinic activities could be safely resumed. -Additional clinician training (~ 2 h) for delivery of SSM and MC via videoconference during COVID restricted period			
**Fidelity**	Planned Fidelity Assessment: Review of structured treatment notes for required, allowed and prohibited intervention procedures and random assessment of video recordings monthly for each provider for 6 months, then quarterly thereafter. If concerns arise, investigators will reinstate monthly fidelity checks and/or additional training as needed.			

BPS = Biopsychosocial; DC = Chiropractor; LBP = Low Back Pain; MC = Medical Care; NSAIDs = Non-Steroidal Anti-Inflammatory Drugs; PT = Physical Therapist; SMT = Spinal Manipulation Therapy; SSM = Supported Self-Management

### Data management and data collection

The DCC supports an https-secured web page (https://pacback.org) that provides a centralized location for public information about the project for potential participants, investigators, and institutional agencies. The web page contains a link to the project portal. Study personnel log on to the private portal on the study web page with individual Shibboleth-based usernames and passwords to securely perform study data management activities. Data collection is conducted using web- and text-based delivery platforms for self-report questionnaires; these are administered free of provider and investigator influence and are overseen and managed by the DCC. Table 3 summarizes the data collection schedule.

**Table 3:** Schedule of Enrollment, Intervention and Assessments

	STUDY PERIOD									
	Eligibility Enrollment & Allocation		Post-allocation							
Timepoint	Initial Screen	Baseline/ Enrollment (Day 0)	Inter-vention phase (Months 0–2)	Weekly Follow-Up (Weeks 1–52)	Follow-Up (Week 2)	Monthly Follow-Up (Months 1–12)	Follow-Up (Month 1)	Follow-Up (Month 2)	Follow-Up (Month 6)	Follow-Up (Month 12)
Informed consent	x	x								
Demographics	x	x								
Medical history & medications		x								
Physical exam including objective outcomes		x						x		
Inclusion/Exclusion criteria	x	x								
Technology assessment**		x								
Intervention administered all 4 arms (booster sessions allowable in month 3–12)			x							
COVID − 19 impact		x						x	x	x
TUQ**		x					x	x		
STarT Back screening tool status	x							x	x	x
Chronic LBP status (NIH research task force definition)	x	x							x	x
Chronic interference with daily activities	x	x							x	x
Low back pain intensity	x	x		x						
Low back pain frequency		x		x						
Pain trajectory		x								x
Implementation measures	x	x						x	x	x
Allocation/Randomization		x								
Intervention uptake								x	x	x
Disability, PROMIS-29, healthcare and medication use, and productivity loss		x				x				
Adverse events*		x	x			x				
Satisfaction and global improvement								x	x	x
Healing Encounters and Attitudes List (HEAL) non-specific factors		x			x		x	x	x	x
Psychosocial mediators (self-efficacy, coping, kinesiophobia, and pain catastrophizing)		x						x	x	x
Participant close out										x

*Participants can also report adverse events to the PI’s or study staff at any point during the trial

** Technology Assessment and the Telehealth Usability Questionnaire (TUQ) will be administered to participants who are enrolled in the 2-arm study only. Tech Assessment may be administered to participants in the 4-arm study if applicable (e.g., preparing for a virtual SSM session)

To provide incentive for complying with follow-up questionnaires, participants receive a small monetary compensation for each of the monthly questionnaires completed. If participants choose to drop out of the trial or discontinue completing the required questionnaires, attempts are made to reach an agreement with participants to fill out at least monthly questionnaires at two-, six-, and 12-months post-randomization.

**Baseline Measures** include demographic, occupational, and clinical data, measured using the adapted acute/subacute version of the National Institutes of Health’s Research Task Force (NIH RTF) minimum dataset [9]. The Quebec Task Force’s classification system for spinal disorders is used for diagnostic classification [62]. Baseline duration of LBP (acute: <6 weeks vs. subacute: 6–12 weeks) and risk of cLBP (STarT Back screening tool status medium vs. high risk) are collected for pre-specified subgroup analyses (described below).

**Adverse Events and Serious Adverse Events (AEs/SAEs)** are identified during the intervention phase at intervention visits; during the study follow-up phase using monthly self-report questionnaires and by participants reporting to study staff. Events are followed until resolution or stabilization, whichever occurs first; resolution and stabilization are determined by the PI with input from a study clinician when appropriate. SAEs potentially related to treatment are brought to the attention of the IRB and the DSMB in writing. As part of the Data Safety and Monitoring Plan (DSMP) the DCC performs continuous and interim analysis of accruing safety data. Potentially treatment-related SAEs are monitored throughout the course of the study. The following guidelines are used when considering halting the trial for safety: [1] > 5% of participants experience an unexpected, related, moderate or greater adverse event; and [2] ≥ 2% SAE overall that are unexpected and related to the intervention. The DSMB considers this guidance when making recommendations regarding trial continuation.

#### Effectiveness measures

The trial has three primary outcome measures: 1) average pain intensity over 12 months post-randomization (0–10 numerical rating scale (NRS)); [63–65] [2] average disability over 12 months post-randomization (0–24 scale, Roland Morris Disability Questionnaire (RMD));[66, 67] and [3] prevention of cLBP that is impactful at 10–12 months follow-up (8–50, LBP Impact scale using mean from months 10–12). The LBP impact scale includes measures of pain intensity, pain interference, and physical function from the PROMIS-29 Profile v2.0) [9].

Secondary outcomes include recovery at 6 months (binary composite outcome defined as pain NRS = 0 and RMD < = 2.) and PROMIS-29 Profile v2.0 measures to assess pain interference, physical function, anxiety, depression, fatigue, sleep disturbance and the ability to participate in social roles and activities [68]. Other secondary outcomes include LBP frequency, over-the-counter and prescription medication use (including class and frequency); healthcare utilization (e.g., MRIs, injections, hospitalizations, surgery, integrative and complementary treatments); productivity loss (e.g., missed work, reduced productivity while at work); [69] STarT Back screening tool status; [54] patient satisfaction; [70] global improvement; [71] chronic LBP status; [9] frequency of LBP interference with daily activities; self-reported LBP trajectory; [72] adverse events and COVID-19 impact. Objective measures included as secondary outcomes are the Quebec Task Force Classification, Timed Up & Go Test, the Sit to Stand Test, and the Sock Test. All objective measures will be assessed by clinicians blinded to the patients’ intervention assignment [62, 73–75].

**Psychosocial Mediator Measures** that are likely to change as a result of treatment, and potentially affect the primary outcomes are also collected. These include self-efficacy (Chronic Pain Self-Efficacy Scale); [76] coping (Coping Strategies Questionnaire); [77] kinesiophobia (Tampa Scale for Kinesiophobia-11); [78] and catastrophizing (Pain Catastrophizing Scale) [79]. All of these measures have been used in clinical research with diverse population including LBP.

**Implementation Measures** are addressed to explore factors that could assess results interpretation and affect future implementation of the experimental interventions. as guided by the Reach, Effectiveness, Adoption, Implementation, and Maintenance framework (RE- AIM) [52, 80]. The RE-AIM framework was developed to help balance the focus of internal and external validity and is an ideal complement to hybrid trial designs. It advocates mixed-methods data collection to gather a diverse range of contextual data from multiple levels of stakeholders. In this trial, quantitative and open-ended survey questions, individual interviews, activity and process tracking are used to assess participants and non-participants (e.g. those not enrolled in the trial), providers and other stakeholders to gather information on demographics, processes, and views related to barriers and facilitators. These measures are summarized in Table 4 and will be addressed in further detail in a separate publication.

The timing and frequency of data collection for outcomes, mediating measures, and participant-level implementation measures are detailed in Table 3. Provider and other stakeholder implementation data is collected throughout the trial’s life-cycle (see Table 4).

**Table 4:** Implementation Data Collection by Stakeholder Level Guided by the RE-AIM Framework

Reach (Proportion and representativeness of individuals willing to participate)	Participants: % excluded by reason; % who participate; comparison of clinical and demographic characteristics of participants versus non-participants; barriers and facilitators* to participation, reasons for declining participation
Effectiveness (The influence of an intervention on important outcomes, including potential negative effects, quality of life, economic outcomes)	Participants: See Effectiveness Outcomes; Healing Encounters and Attitudes List (HEAL) patient-provider connection, healthcare environment, treatment expectancy, and positive outlook; [81] COVID-19 Impact^+^; [82]) Telehealth Usability Questionnaire (TUQ)^+^; [83] % drop out of treatment in short term (2 months) by patient characteristics, intervention; barriers and facilitators*to intervention effectiveness
	Providers: confidence in own pre-defined intervention related competencies (0–10); modified TUQ^+^; confidence in remote environment^+^; [84] barriers and facilitators*to intervention effectiveness
Adoption (The proportion and representativeness of intervention staff willing to initiate and adopt the intervention)	Providers: demographics, training/educational background; Pain Attitudes and Beliefs Scale (PABS) [85]
Implementation (How consistently various elements of an intervention are delivered as intended)	Participants: adaptations to interventions; barriers and facilitators* to engaging in interventions at 2 months; % of prescribed visits attended (fidelity)
	Providers: adaptations to intervention delivery processes; % of required intervention elements delivered (fidelity); barriers and facilitators* to delivering interventions
	Other staff, system stakeholders: estimated costs, time and resources needed to implement intervention with fidelity; opinions about process**
Maintenance (The extent to which participants make & maintain a behavior change and the sustainability of a program)	Participants: % completed and % lost to follow up at 2, 6 and 12 months; % return to baseline pain and disability; % change in primary outcomes (see Responder Analysis); barriers and facilitators* to engaging in intervention recommendations at 6 and 12 months
	Providers: changes in attitudes and beliefs after trial completion; views of barriers and facilitators* to implementing intervention in the field

*Barriers and facilitators assessed qualitatively; ^+^ Measures added during COVID-19

Patient implementation measures assessed at initial screen through follow up (see Table 3); Provider implementation measures assessed pre-/post- training and at trial completion; **Other staff and system stakeholders assessed throughout the study and in a post-trial qualitative process evaluation

### Sample size and power

The trial focuses on two separate effectiveness research questions with different time frames. First, we consider the overall time-averaged patient status for both pain and disability over the full 12 months of follow-up. We recognize the importance of considering multiple comparisons when evaluating both average pain and average function over one year, and we therefore adopt a multiple comparison correction for these two outcomes. Second, we consider the long-term impact of LBP using an assessment of impact over months 10–12. However, given the different focus of the major questions and the timing of assessments further adjustment for multiple comparisons across the two questions is not indicated. Sample size and power for the trial is based on these two effectiveness research questions. To characterize the power of our primary analyses (an overall ANOVA F-test) a summary table is provided that considers potential standardized mean differences comparing the individual intervention arms to medical care (see Table 5). For a small effect size (Cohen’s d of 0.2) and an additive effect we have greater than 88% power to reject the null. However, additivity may not hold so we also consider sub-additive scenarios, in addition to scenarios where only one intervention is effective. For small to moderate effect size differences (0.25–0.35) we have > 80% power for all scenarios for LBP impact and most scenarios for pain and disability. We assume n = 1000 enrolled with 90% follow-up that yields 900 evaluated participants. Power is based on 5000 simulations per scenario with adjustment for period (4-arm, 2-arm) and site, and accounting for the group imbalance that results from our temporary restriction to 2-arm randomization (see trial modifications due to Covid page 11).

**Table 5:** Standardized mean differences relative to medical care and power

Scenario	SMT Alone	SSM Alone	SSM + SMT	Power	
				Average pain intensity & disability over 12 months post-randomization (alpha = 0.05/2)	Chronicity based on LBP impact averaged over months 10–12 (alpha = 0.05)
*Additive*	0.20	0.20	0.40	88%	93%
*Sub-Additive 1*	0.25	0.25	0.35	85%	91%
*Sub-Additive 2*	0.30	0.30	0.30	89%	94%
*Sub-Additive 3*	0.25	0.25	0.25	74%	81%
*Single effect 1a*	0	0.30	0.30	96%	98%
*Single effect 1b*	0.30	0	0.30	88%	94%
*Single effect 2a*	0	0.25	0.25	83%	91%
*Single effect 2b*	0.25	0	0.25	71%	80%

LBP = Low Back Pain; SMT = Spinal Manipulation Therapy; SSM = Supported Self-Management

### Statistical analysis plan

#### Effectiveness analyses

##### Primary outcomes analyses

Average pain over 12 months post-randomization, average low back disability over 12 months post-randomization and prevention of cLBP that is impactful (based on averaged LBP Impact scores from the PROMIS-29 Profile v2.0 over months 10–12), will be analyzed using a single ANOVA with an omnibus test for the equality of means across the four treatment groups. Linear regression with adjustment for site, baseline risk group, and study period (4-arm, 2-arm) will be computed separately for each outcome. Because pain and disability are measured within the same timeframe, we will apply multiple comparison correction for the two outcomes (using the Holm-Bonferroni method as detailed in FDA guidance); primary estimation contrasts are SMT versus MC, SSM versus MC, and SSM + SMT versus MC using Fisher’s least significant difference. All group comparisons of the primary outcomes will be presented as mean differences with 95% confidence intervals. A secondary evaluation will consider whether the effects of SMT and SSM are potentially synergistic or antagonistic and will be done using a formal test for interaction.

#### Additional analyses of primary outcomes

*Responder analyses*. We will conduct responder analyses by assessing the proportion of patients experiencing ≥ 50% improvement in pain or disability from baseline to six months, and from baseline to twelve months. We will also evaluate the proportion experiencing ≥ 30% improvement, and conduct a comprehensive responder analysis that looks at the cumulative percentage of participants achieving a range of improvement [86].

*Longitudinal analyses*. We will use the monthly measures of disability and weekly measures of pain to conduct longitudinal analysis that characterizes the mean profile over time for each intervention group. Formal comparison of profiles will be based on linear mixed models or generalized estimating equations (GEE). We will also report on changes from baseline to post treatment at 2 months and at 6 and 12 month follow-ups. These analyses will evaluate the magnitude of short, medium, and long-term effects of treatment, which are traditionally used in systematic reviews and meta-analyses. Furthermore, we will conduct exploratory analyses that assume latent classes with associated trajectories, and we can evaluate whether these groups differ across the intervention arms [87, 88].

##### Missing data

Missing data may include missing covariate information, study dropout, or missed and/or mistimed participant visits. While the protocol includes procedures to ensure the most “complete” follow-up data on every enrolled participant, it is likely that some participants will have incomplete data. We will determine reasons for missingness and classify each missingness pattern as missing completely at random (MCAR), missing at random (MAR), or missing not at random (MNAR). The MCAR mechanism occurs when the probability of response is independent of both the observed data and the unobserved data [89]. In longitudinal analyses, likelihood-based analyses of complete-case data for the linear mixed-effects model, the generalized linear mixed-effects model, and the nonlinear mixed-effects model lead to valid inference under MCAR and MAR mechanisms, whereas the GEE analyses lead to valid inference only in the presence of MCAR mechanisms [89, 90]. Statistical tests to assess the validity of the MCAR assumption in certain circumstances are available, but they are model-dependent and non-robust [91–93]. In general, we will advocate the use of multiple imputation (MI) [94] both to assess the sensitivity of results and to correct for potential bias from missing covariates. We will consider the missing data mechanism, analysis approach, and plausibility of the congeniality assumption [95, 96].

*Subgroup/Moderator Analyses*: We will perform two pre-specified subgroup analyses to look at treatment effects within: risk of cLBP based on the STarT Back (medium vs. high); and participants stratified based on their duration of LBP (acute vs. sub-acute). Subgroup analyses will use linear regression among restricted subsets to quantify specific treatment effects, and formal evaluation of differences in treatment effects across subgroups will be conducted using treatment by subgroup interactions.

##### Mediation analysis for psychosocial factors

Formal mediation analysis [97–99] will focus on characterizing the degree to which self-efficacy, coping, kinesiophobia, and pain catastrophizing measured at 8 weeks can explain treatment effects at 6 months, and whether these measures obtained at 6 months explain long-term treatment effects (1 year). We will quantify the percent of the treatment effect that is explained by changes in each scale individually, and in totality when included in a multivariate model for the outcome [100]. We will analyze mediation for LBP impact, pain NRS, and RMD measured at 6 months and 1 year.

##### Secondary outcomes analyses

All secondary outcomes, except the recovery outcome, will be assessed for each of the three intervention groups (SMT, SSM and SSM + SMT) relative to MC. We will use linear mixed models or GEE for longitudinal analysis [101]. We will also report on changes from baseline to post treatment at 2 months and at 6 and 12 months follow- up. These time-point analyses will evaluate the magnitude of short, medium, and long-term effects of treatment, for use in systematic reviews and meta-analyses.

The main secondary outcome of recovery at 6 months (a binary composite outcome derived from pain NRS = 0 AND RMD < = 2.) will be analyzed using a test on the overall (marginal) effect of SMT and a test of the overall (marginal) effect of SSM. These tests are obtained using logistic regression including the interaction between SMT and SSM and then overall effects are computed as a linear contrast of the two stratum-specific comparisons (i.e., the overall SMT effect is defined as the average of the SMT effect when SSM = 0 and the SMT effect when SSM = 1). We will assess the impact of alternative definitions of recovery (e.g., NRS < 3 and RMD < 4). We will also consider the time-until-recovery based on measurements taken every 4 weeks during the twelve months of follow-up. Specifically, we can define the time-until-recovery as the assessment month in which the subject is first observed to achieve an NRS = 0 and RMD < = 2. We will use discrete time (monthly data) cumulative incidence curves to show the percent of subjects in each treatment group who have achieved a first recovery by each follow-up time period. Because recovery may not be maintained, and subjects may subsequently relapse, we will display plots showing the percent of subjects who are currently in the recovered state as a function of time. Formal comparison of cumulative incidence curves can be obtained using the log rank test since in this situation the cumulative incidence is simply 1-survival as would be computed using Kaplan-Meier curves. In addition, we will use a model-based survival analysis.

The robustness of NIH RTF case definition of chronic LBP will be assessed using measures of pain frequency and LBP-related burden (pain, disability, productivity loss, healthcare utilization) by assessing differences between participants meeting the case definition and those who do not.

### Implementation analyses

Quantitative data will be analyzed using descriptive statistics, independent t-tests (for means) and z-tests (for proportions) to assess group differences as appropriate. For the qualitative analysis, teams of 2–3 will perform rapid deductive, directed content analytic methods; the coding structure and operational definitions will be guided by the study’s conceptual models to provide insights into barriers and facilitators to future implementation [51, 58, 59]. Directed content analyses will also allow for inductive gathering of important themes that might fall outside of our chosen models and frameworks [102, 103]. Rapid approaches have been advocated for implementation research as they balance rigor with efficiency, yielding timely and meaningful evaluation of stakeholder needs and perspectives that can be more quickly matched to solutions [102, 104].

Additional Statistical Considerations: A priori criteria were established for combining study data from the UG3 and UH3 phases for the statistical analysis. NCCIH, and the DSMB reviewed and approved combining data for the UG3 and UH3 phases at the end of the UG3 phase.

### Dissemination

A Publications Committee with representation from the CCC and the DCC facilitates timely dissemination of study findings, maintains high scientific standards for published material, prioritizes the order of publication and presentations, and ensures equitable investigator participation and attribution of authorship. The committee ensures publications are well-aligned with the trial’s research objectives and are not redundant. The committee also reviews and guides all data analysis plans, as well as research abstracts, presentations, and manuscripts before submission. The committee reviews proposals for ancillary studies and ensures all publications meet the NIH Open Access criteria including deposit in PubMed Central.

### Protocol modifications and impact on the trial

All significant protocol modifications were reviewed and approved by NIH, the DSMB, and IRB.

**Change in Eligibility Criteria.** Our definition for an acute/sub-acute episode of LBP was re-examined and changed during the UG3 phase of the trial due to difficulty recruiting patients. Our initial definition required a one-month period without bothersome LBP prior to the start of the episode. In addition, participants were required to report LBP interfered with their regular daily activities on less than half of the days over the past six months. We encountered many individuals with ongoing back problems that had experienced a recent worsening or aggravation for which they were seeking care but were ineligible. In addition, many patients had difficulty interpreting what constituted “bothersome LBP symptoms” and recalling LBP symptoms accurately over the past six months. As a result, we updated the definition to include aggravations of LBP using a recommended definition from an international group of LBP experts [53]. To qualify, participants must experience a new episode or aggravation of ongoing LBP in the past 12 weeks that lasted at least 2 weeks. Further, individuals with an aggravation of their LBP must agree that it is a worsening of their condition that is difficult to tolerate and generally impacts their usual activities and/or emotions. We also updated the requirements in the month prior to the new episode/aggravation from “no bothersome LBP symptoms” to “less than severe LBP on average” and dropped the requirement based on symptom recall in the past 6 months. This change enabled a more accurate identification of eligible participants without biasing the trial.

### Change in primary outcome for preventing chronic LBP

Our original primary outcome for preventing chronic LBP used the NIH RTF items based on participant recall for the frequency of LBP in the past six months with LBP on half the days or more considered chronic. The NIH RTF chronicity outcome measure has several recognized limitations. The measure is based on a 6-month recall period and was not intended to be used as an outcome measure by the NIH RTF (April 2021 personal communication with RTF member and co-investigator of this trial, Dennis Turk and chairman of the RTF, Rick Deyo). Further, it is a dichotomous outcome that does not consider the severity of pain or the amount of interference with daily activities and therefore does not define the degree of overall impact. After the trial began, new information highlighting the importance of measuring the impact of chronic pain was published [6–8, 10, 11]. This recent body of literature prompted us to reevaluate the adequacy of our primary chronicity outcome measure. We decided to adopt the chronicity measure that was already being collected at a monthly basis, prevention of impactful cLBP (based on averaged LBP Impact scores from the PROMIS-29 Profile v2.0 over months 10–12) [9].

By transitioning from a dichotomous to a quantitative primary outcome, we gained statistical power to evaluate the LBP impact/chronicity aim. Based on our updated power calculations, the impact of imbalance in the randomization allocation due to restricted randomization was minimal. By reducing the original sample size of 1180 based on the recovery outcome to 1000 participants we retain excellent power for determining clinically meaningful group differences on all primary outcomes with effect size differences of at least 0.30.

### Change in statistical analysis plan and effectiveness objectives to control for multiplicity

The trial initially had three main effectiveness objectives: (1) prevention of chronic LBP at twelve months; (2) recovery from acute/sub-acute LBP at six months; (3) Average of pain and disability over twelve months. In 2021, the NIH statistician overseeing the trial raised the question of the adequacy of the planned adjustment for multiplicity given the trial’s three main effectiveness objectives and accompanying four co-primary outcome measures. In response to this concern, the lead investigators recommended the recovery objective be changed to a key secondary outcome. Early in the conduct of the trial it was decided and approved as a protocol change to include patients that had an acute aggravation of ongoing LBP, if the ongoing pain was not rated as severe in the month prior to the aggravation. This protocol change substantially lowers the proportion of patients that can be expected to recover according to our criteria (pain severity = 0 and RMD score ≤ 2). Given this change, the recovery outcome was less appropriate as a primary effectiveness objective and demoting it to a secondary outcome mitigates the concern of cross-objective control. To further mitigate concerns of cross-objective control for multiplicity, the investigative team developed a publication plan that addresses two separate research questions that would normally be the focus of two separate trials. The first manuscript will focus on the primary outcomes listed for the cumulative LBP and disability experience over twelve-months post-randomization. This paper would present recovery at six months as the key pre-specified secondary outcome. The second manuscript will focus on chronic impact at twelve months using the pre-specified primary outcome of LBP Impact averaged over months 10–12. The focus of the second manuscript will have a different objective from the first and would not warrant adjustment for multiple comparisons as the separate endpoint, distinct twelve-month outcome time period, and separate publication all imply no correction for other analyses.

### Extenuating circumstances and impact on the Trial

**COVID-19 Pandemic**: In March 2020, the COVID-19 global pandemic resulted in a temporary suspension of the trial, including recruitment, enrollment, intervention delivery, and the collection of objective secondary outcomes. In response to increased severity of the COVID-19 pandemic, including increased rates of community spread, hospitalization and death rates and a rapidly changing environment, we made important modifications to the trial protocol. Two of the trial arms (SMT and SSM + SMT) required face-to-face contact with study participants. In order to avoid physical interaction, we updated the protocol to allow for remote assessments and interventions in a partial 2 group randomization period during which participants were randomized only to MC or SSM, delivered using HIPAA-compliant videoconferencing technology. In order to accomplish this, several modifications were made including: transitioning to an electronic consent process; updating study protocols and training staff to assess eligibility criteria, deliver SSM and MC, and objective measures via telehealth; addition of secondary outcome measures regarding COVID-19 impact and telehealth usability; modifications to randomization scheme for the partial 2-group randomization period; and implementation of active COVID-19 monitoring at participating sites. The partial 2-group randomization period began in December 2020. In November 2021, when conditions were met to safely return to in-person activities, we returned to full 4 group treatment allocation. Clinic activities that were suspended at UM Epidemiology Clinical Research Center due to COVID-19 were moved to the Berman Center for Outcomes and Clinical Research. In order to account for potential period-effects, we updated our statistical analysis plan to include an adjustment for partial randomization time periods in all analyses. Since November 2021, all trial procedures have been compliant with Covid mitigation rules (masking, distancing, and sanitation of surfaces) established by the Universities of Minnesota and Pittsburgh. All modifications were planned by the principal investigative team, reviewed and approved by the DSMB and the funding agency, and reported within the trial registration at ClinicalTrials.gov.

**George Floyd Murder & Social Unrest in the Twin Cities (Minneapolis-St Paul)**. In May 2020, George Floyd was killed by a uniformed Minneapolis police officer only a short distance from where study activities normally took place. Mr. Floyd’s murder resulted in considerable trauma and social unrest in the Twin Cities, including riots, shootings, vandalism and looting. The impacts of this continue to affect communities of color. The original recruitment plan included planned events and initiatives to recruit diverse populations. However, the study team was advised by community partners that participation in research during these troubling times simply wasn’t a priority, and we were encouraged to be sensitive to the communities’ needs. As a result, the study team made important modifications to the original recruitment plan at the advice of community study consultants.

## Discussion

This trial aims to fill an important gap in the scientific literature regarding the effectiveness of promising non-pharmacological treatments compared to medical care in the management patients with an acute episode of LBP and the prevention of progression to severe cLBP, by targeting those at higher risk [23].

**Fig. 2:**
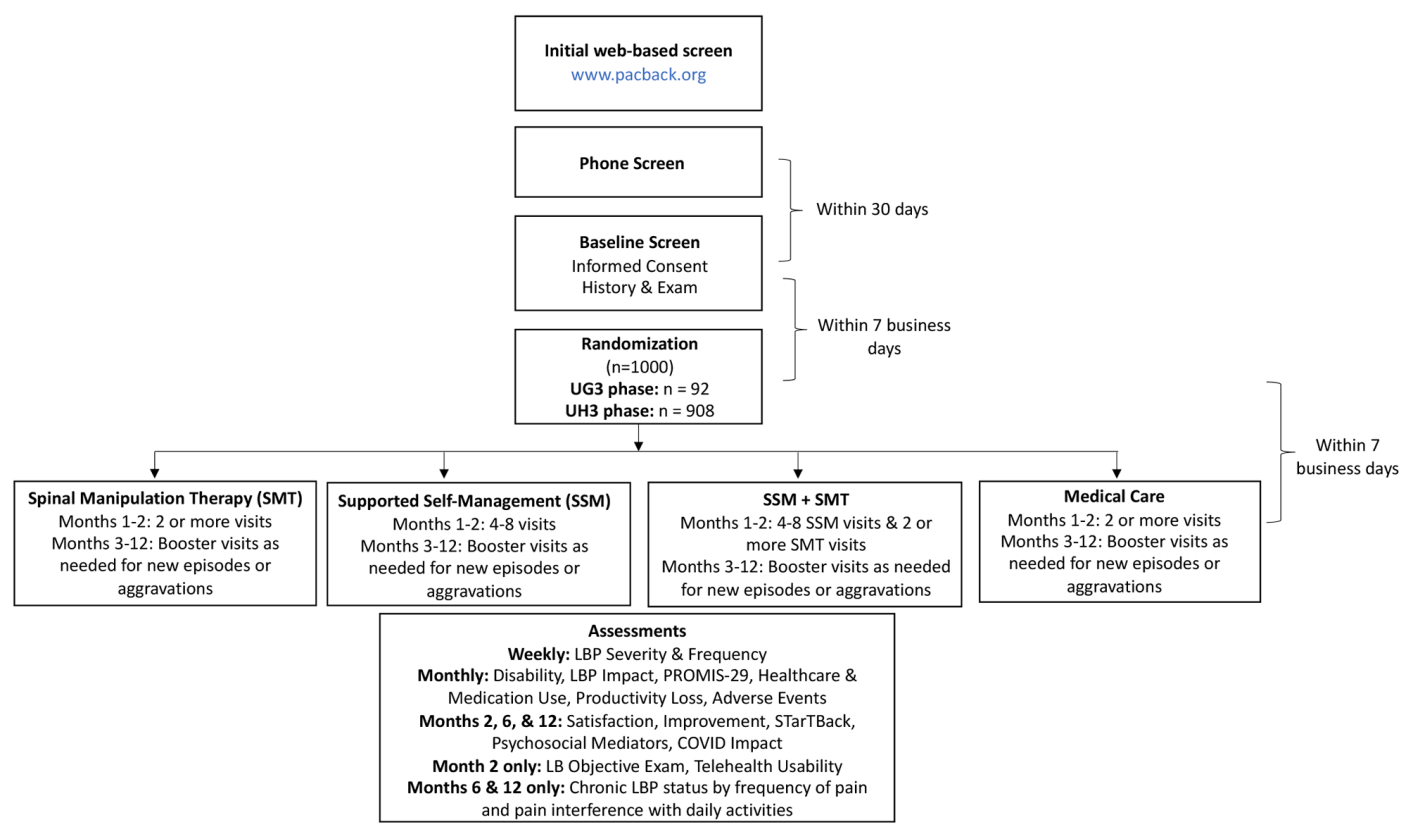
Trial flow diagram

The trial has several strengths that will facilitate the advancement of LBP research and practice. First, the hybrid effectiveness implementation design was informed by the PRECIS tool, [52] which maximizes pragmaticism while including several explanatory elements to ensure internal validity. Noteworthy pragmatic design features include outcomes that span biopsychosocial domains relevant to patients with pain and a high degree of intervention flexibility (see Fig. 2) including tailoring and individualization to meet patient needs (see Table 2). Further, while type 1 hybrid designs prioritize effectiveness outcomes, the trial also uses the RE-AIM translational framework to consider key contextual factors and processes from different stakeholders that can facilitate or impede eventual intervention implementation [51, 105]. While such approaches have gained traction in other health fields [106–108] for addressing the translation of effective interventions to practice, there have been few such studies in the LBP arena [109].

This trial seeks to overcome some of the limitations of existing research, especially regarding self-management. This includes heterogenous content and format, [110, 111] poor methodological quality, lack of long-term follow-up, inattention to intervention fidelity and absence of theoretical rationales aligning patient needs and risk factors [112]. We have used the TIDieR checklist to guide the reporting of the interventions to facilitate future results interpretation, as well as dissemination and replication [61]. Additionally, in accordance with reporting standards, we have described protocol modifications with their potential impacts, including those due to extenuating circumstances which were beyond the research team’s control [32].

Another strength of this research is the application of theoretical frameworks to the experimental intervention design (SSM and SSM + SMT). We used the well-established behavior change wheel (BCW) model, coupled with the dynamic biopsychosocial model of pain that acknowledges the complex and reciprocal interactions between the evolving biopsychological or “whole” person and their external, social environment [57–59]. While widely applied to other health conditions, the BCW has rarely been applied in LBP research. An advantage of the BCW is that it represents a synthesis of 19 behavioral theoretical frameworks and thus is more comprehensive in addressing the complexity of human behavior versus a single theory driven model. The application of the BCW in the intervention design, as well provider training, will be addressed in depth in a subsequent publication [59, 113].

Also noteworthy is the pragmatic advantage to having the biopsychosocial elements of care delivered by a single practitioner in the SSM and SSM + SMT interventions. This has the potential to improve patient access to harmonized, multi-modal care and decrease patient burden and associated costs [114]. PTs and DCs are the most common providers of non-pharmacologic treatment for back pain conditions in the United States [27]. This makes them optimally positioned for delivering integrated psychosocial strategies to complement biological/physical approaches, [26, 28] and play a critical role in the frontline non-drug management of LBP [29, 30]. Indeed, there have already been shifts in both the PT and DC fields to integrate more psychosocial aspects into their care models to better support patient self-management [26, 28, 115, 116].

## Data Availability

Study materials are available from the corresponding author by reasonable request.

## Abbreviations

AE: Adverse Event
BCW: Behavior Change Wheel
BPS: Biopsychosocial
CCC: Clinical Coordinating Centers
cLBP: Chronic Low Back Pain
DCC: DC Chiropractor
DSMB: Data Safety and Monitoring Board
DSMP: Data Safety and Monitoring Plan
GEE: Generalized Estimating Equations
HIPAA: Health Insurance Portability and Accountability Act
IRB: Institutional Review Board
LBP: Low Back Pain
MAR: Missing at Random
MC: Medical Care
MCAR: Missing Completely at Random
MNAR: Missing Not at Random
NCCIH: National Center for Complementary and Integrative Health
NIH: RTF National Institute of Health’s Research Task Force
NRS: Numerical Rating Scale
NSAIDs: Nonsteroidal Anti-inflammatory Drugs
PACBACK: Preventing Acute to Chronic Back Pain
PRECIS: Pragmatic Explanatory Continuum Indicator Summary
PT: Physical Therapist
RE-AIM: Reach, Effectiveness, Adoption, Implementation, and Maintenance
RMD: Roland Morris Disability Questionnaire
SAE: Serious Adverse Event
SMT: Spinal Manipulation Therapy
SSM: Supported Self-Management
TIDieR: Template for Intervention Description and Replication
UM: University of Minnesota
UP: University of Pittsburgh
UW: University of Washington
